# Synthesis of Prussian Blue-Containing Polymeric Nanocapsules via Interfacial Confined Coordination in Crosslinked Miniemulsion

**DOI:** 10.3390/nano16090541

**Published:** 2026-04-29

**Authors:** Lin Wu, Yubin Zhou, Tao Pang, Laxia Wu, Yebin Guan

**Affiliations:** 1 Anhui Ultra High Molecular Weight Polyethylene Fiber Engineering Research Center, School of Chemistry and Chemical Engineering, Anqing Normal University, Anqing 261433, China; y24160011@stu.aqnu.edu.cn; 2 Anhui Provincial Key Laboratory of Advanced Catalysis and Energy Materials, School of Chemistry and Chemical Engineering, Anqing Normal University, Anqing 261433, China; 160067@aqnu.edu.cn (T.P.); wulaxia@aqnu.edu.cn (L.W.)

**Keywords:** miniemulsion, crosslinking, Prussian Blue, polymeric nanocapsule, hybrid nanomaterial

## Abstract

Herein, we describe a versatile synthetic strategy for constructing Prussian Blue (PB)-coated polymeric nanocapsules (PB@nanocapsules) with tunable sizes and controlled PB loading. A soft template was first formed from a miniemulsion composed of water/chloroform/hexadecane (94.55:5:0.2, *w*/*w*/*w*), using P4VP_82_-*b*-PDMAA_180_ as a stabilizer and varying amounts of P4VP homopolymer as a hydrophobe and additional reactive site provider. Crosslinked nanocapsules were obtained by adding 1,2-bis-(2-iodoethoxy)ethane (BIEE) as a crosslinker. The resulting nanocapsules exhibited average hydrodynamic diameters ranging from approximately 282 nm (without P4VP homopolymer) down to 58 nm (with 0.01 g P4VP homopolymer), as determined by DLS and TEM. Subsequently, sequential coordination with sodium pentacyanoammine -ferroate(II) hydrate (Na_3_ [Fe(CN)_5_NH_3_]), followed by the addition of FeCl_3_, yielded a uniform PB coating, as confirmed by the appearance of a characteristic absorption peak at 780 nm in the UV–Vis spectra and a CN stretching shift from 2060 to 2070 cm^−1^ in FT-IR. TEM and HAADF-STEM with EDX mapping revealed the homogeneous distribution of Fe across the nanocapsule shells. The PB loading could be further controlled by varying the Fe^3+^ addition (5.0 × 10^−3^–4.5 × 10^−2^ mmol), with higher loading improving thermal stability. This rational design provides a robust and generalizable platform for engineering polymer–inorganic hybrid nanostructures with tailored functionalities.

## 1. Introduction

Polymer/inorganic hybrid nanomaterials have garnered significant interest in scientific and technical fields owing to their ability to leverage the performance advantages offered by both inorganic and polymeric materials. The unique properties of hybrid nanomaterials—including their substantial stability, high biocompatibility, and adaptable flexibility—can be leveraged to address the requirements of a broad spectrum of applications [1,2,3,4,5].

Prussian Blue (Fe_4_ [Fe(CN)_6_]_3_•xH_2_O) is a mixed-valence coordination polymer comprising alternating Fe(II) and Fe(III) centers bridged by cyanide ligands. It possesses a cubic lattice structure with the [Fe(CN)_6_]^4−^ unit in an octahedral coordination geometry, and its characteristic deep blue color arises from intervalence charge transfer between Fe(II) and Fe(III) [6]. Owing to its unique mixed-valence structure, Prussian Blue has been widely explored in various fields [7,8,9]. To date, polymer–Prussian Blue (PB) nanomaterials represent a class of highly studied polymer–inorganic hybrids, defined by their magnetic, conductive, and catalytic capabilities [10,11,12,13,14,15]. Current efforts have resulted in the development of several techniques for creating polymer–PB nanostructures with various morphologies, such as sphere-like structures [14,16,17,18,19], cubic-like shapes [20,21,22], and others [23,24]. This morphological control is primarily achieved by employing polymers as soft templates, which guide PB growth through the formation of regulatory structures. Wang and co-workers reported the fabrication of uniform polymer–PB nanospheres by using a micellar templating approach. Pentacyanoferrate-coordinated P(4VPFe-co-4VP)-*b*-PDMA block copolymers first self-assemble into micelles in H_2_O/EtOH solvent, which then serve as confined reactors for the in situ formation of PB nanoparticles upon the addition of FeCl_3_ [19]. Similarly, Marty and co-workers employed a polymer-directed templating strategy to fabricate hybrid PB nanocubes. In their approach, poly(ethylene oxide)-block-poly(acrylic acid) (PEO-*b*-PAA) first coordinates with Fe^3+^ in aqueous solution, forming a polymeric complex [22]. The subsequent addition of potassium ferrocyanide then induces the confined growth of PB within this template, yielding nanocube structures. Notably, the size of the resulting nanocubes could be precisely tuned by varying the pH of the reaction medium, either conventionally via acid/base addition or dynamically through the use of a photoacid.

Beyond solid spheres and cubes, hollow-structured Prussian Blue possesses a larger specific surface area and demonstrates excellent performance in areas such as catalysis, drug delivery, active factor loading, and electrochemical storage [2,15,25,26,27,28]. Yamauchi et al. employed a chemical etching method to synthesize nanosized PB with hollow interiors and good crystallinity. The method employs PB mesocrystals as the starting material, which are formed by the oriented aggregation of PB nanocrystals and exhibit single-crystal-like behavior. Through diffusion of the etching solution into the pores or defects between the nanocrystals within the mesocrystals, an interior hollow structure has been successfully achieved while preserving the original crystallinity of PB [29]. However, this “hard-template method” for synthesizing hollow Prussian Blue structures involves the chemical etching of the sacrificial template cores. Usually, the selection of strong acids for etching makes the procedure both risky and complex [30,31]. Compared to the hard-template method, the use of microemulsion or miniemulsion as a soft template offers a distinct advantage. The hollow interior of the polymer-based vesicle structure can be easily obtained through simple water washing or demulsification once the vesicles are formed, thereby eliminating the need for specialized or complex equipment. The Wang research group first reported the miniemulsion periphery polymerization (MEPP) method to prepare PB@polymer hollow spherical nanoparticles by using the miniemulsion method [16]. They employed a pentacyanoferrate-terminated PEG-PPG-PEG triblock copolymer to form the miniemulsion. Subsequently, the addition of Fe^3+^ ions triggered metal coordination at the droplet interface, leading to the formation of the hollow composite structure with a rigid shell. Subsequently, Wang et al. successfully synthesized Prussian Blue coordination polymer nanoboxes with a rigid shell using the MEPP method under benign thermal and chemical conditions [32]. However, the process requires the prior preparation of the metallo-surfactant, such as pentacyano(4-(dimethylamino)pyridine) -ferrate-terminated poly(ethylene glycol)-*b*-poly(propylene glycol)-*b*-poly(ethylene glycol) (EPE-Fe), which is a highly tedious step. In addition, the synthesis resulted in a rigid shell structure, and the inherent flexibility of the polymer was not exhibited within the system.

In our previous study, we developed a technique for the preparation of polymeric nanocapsules using poly(*N*,*N*-dimethylaminoethyl methacrylate) (PDMAEMA) homopolymers or poly(*N*,*N*-dimethylaminoethyl methacrylate)-block-poly(butyl methylacrylate) (PDMAEMA-*b*-PB) block copolymers as stabilizers in a miniemulsion [33,34]. We found that these polymers could be easily crosslinked by 1,2-bis-(2-iodoethoxy) ethane (BIEE) to form soft polymeric nanocapsules under mild conditions. Similarly, by utilizing BIEE as a crosslinker, the nitrogen atoms on the pyridine group of poly(4-vinyl pyridine) (P4VP) can be selectively quaternized and crosslinked [35,36]. In this work, we report a novel and facile method for the preparation of soft-shell Prussian Blue–polymer composite nanocapsules. Poly(4-vinyl pyridine)_82_-block-poly(*N*,*N*-dimethylacrylamide)_180_/P4VP_82_-*b*-PDMAA_180_ was utilized as a crosslinkable stabilizer in miniemulsion preparation. The P4VP segment could potentially be crosslinked to form a polymer shell structure. The unreacted P4VP can undergo further reaction with Na_3_ [Fe(CN)_5_HN_3_], leading to the in situ generation of PB on the polymer capsule through the continuous addition of FeCl_3_ to the system. This approach offers several key advantages over existing methods: (1) it utilizes a soft-template miniemulsion technique, enabling the convenient preparation of vesicle structures with tunable dimensions without the need for harsh etching processes; (2) it achieves the in situ generation of Prussian Blue on a pre-formed polymer capsule, eliminating the need for pre-synthesized metallo-surfactants; and (3) it preserves the inherent flexibility of the polymer matrix, resulting in composite capsules with a unique soft-shell architecture. These soft-shell Prussian Blue–polymer nanocapsules hold significant promise for applications in controlled drug release, bioimaging, heavy metal adsorption, and energy storage, offering new design strategies for biomedical applications requiring both flexibility and functionality.

## 2. Materials and Methods

### 2.1. Materials

4-vinylpyridine (4VP, ≥96%) and *N*,*N*-dimethylacrylamide (DMAA, ≥99%) from Aladdin (Shanghai, China) were purified via passage through an alumina column prior to polymerization. Sodium pentacyanoammineferroate(II) hydrate was obtained from Tokyo Chemical Industry (Tokyo, Japan); hexadecane (HD) was from Alfa Aesar (Ward Hill, MA, USA); iron(III) chloride hexahydrate (FeCl_3_•6H_2_O) was from Aladdin; 2-cyano-2-propyl benzodithioate (CPDB, >97%) and 1,2-bis(2-iodoethoxy)ethane (BIEE) were from Sigma-Aldrich (St. Louis, MO, USA). All these chemicals were used as received. The initiator 2,2′-azobisisobutyronitrile (AIBN, ≥98%, Aladdin (Shanghai, China)) was subjected to recrystallization from ethanol prior to utilization.

### 2.2. Characterization

^1^H NMR (400 MHz) measurements were conducted on a JEOL ECA spectrometer (JEOL ECA, JEOL Ltd., Tokyo, Japan) operating at ambient temperature (about 20 °C), utilizing CDCl_3_ for sample preparation. The number-average molecular weight (Mn) and polydispersity index (PDI) of the polymers were measured via gel permeation chromatography (GPC) on a PL-GPC220 system (Agilent Technologies, Santa Clara, CA, USA) equipped with a refractive index (RI) detector. Separation was performed using a GRAMMY linear column (8 × 300 mm, 10 µm; Agilent Technologies) with *N*,*N*-dimethylformamide (DMF) as the eluent at a flow rate of 1.0 mL·min^−1^ and a column temperature of 40 °C. Poly(methyl methacrylate) (PMMA) was used as the calibration standard. Transmission electron microscopy (TEM) characterization was performed using an FEI Tecnai G2 F20 instrument (FEI Company, Hillsboro, OR, USA). Meanwhile, the elemental compositions of the samples were analyzed by energy-dispersive X-ray spectroscopy (EDS) with an Oxford Instruments X-Max 80T detector (Oxford Instruments, Abingdon, UK). AFM imaging was performed on a Bruker Dimension Icon atomic force microscope (Bruker, Billerica, MA, USA). The average size and polydispersity index of versicles were determined using DLS at 25 °C. The sample was analyzed using a Malvern Zetasizer Pro. (Malvern Panalytical Ltd., Malvern, UK). The infrared spectra were recorded from 400 to 4000 cm^−1^ on an iS50FT-IR spectrometer (Thermo Fisher Scientific, Waltham, MA, USA) by using KBr pellets. UV–Vis spectra were recorded on a UV-2501PC spectrometer (Shimadzu Corporation, Kyoto, Japan). Thermogravimetric analysis (TGA) was performed on a TA Instruments TGA-2900 analyzer (TA Instruments, New Castle, DE, USA). The measurements were obtained from 25 to 600 °C at a heating rate of 10 K/min under a nitrogen atmosphere. Zeta (ζ) potential measurement of samples was determined by BeNano Zeta (Bettersize Instruments Ltd., Dandong, China).

### 2.3. Synthesis of Poly(4-vinyl pyridine) (P4VP)

The synthesis of P4VP (target DP = 100) was carried out by bulk reversible addition–fragmentation chain transfer polymerization. A mixture of monomer 4VP (10.0 g, 95.1 mmol), AIBN (0.031 g, 0.189 mmol) and chain transfer agent CPDB (0.210 g, 0.951 mmol) was purged with N_2_ for 20 min and then initiated at 60 °C for 24 h. The reaction was quenched by immersion in an ice-water bath. The crude product was dissolved in THF, precipitated into diethyl ether, and purified via three cycles of precipitation/filtration. After filtration and vacuum drying at room temperature overnight, 8.87 g of a pink polymer was obtained (yield: 86.6%). The resulting product exhibited a number-average molecular weight of 7200 and a molecular weight distribution of 1.24, as determined by GPC.

### 2.4. Synthesis of Poly(4-vinyl pyridine)-block-poly(N,N-dimethylacrylamide) (P4VP-b-PDMAA)

AIBN (0.0067 g, 0.041 mmol) and P4VP_82_ (2 g, 0.226 mmol) were dissolved in 2 mL of DMF, followed by the addition of *N*,*N*-dimethylacrylamide (5.12 g, 0.052 mol). The resulting mixture was deoxygenated via purging with nitrogen gas for 30 min. Polymerization was carried out at 70 °C for 18 h. The reaction was terminated by ice-bath cooling. The resulting product was then precipitated into diethyl ether, and the solid was isolated by suction filtration, yielding 72.6%. GPC using PMMA standards determined the number-average molecular weight Mn as 18,900 g·mol^−1^and the polydispersity index (PDI) as 1.48.

### 2.5. Preparation of Polymeric Nanocapsules

A representative preparation procedure was typically carried out as follows: P4VP_82_-*b*-PDMAA_180_ (0.025 g, 0.938 × 10^−3^ mmol) and hexadecane/HD (20 mg) were dissolved in 0.5 g of chloroform to afford a homogeneous mixture. (Note: In various experimental groups, varying amounts of P4VP homopolymer with a molecular weight of 19,578 g mol^−1^ were selectively added.) This organic phase was then added to DI water (9.455 g) with vigorous stirring to form a coarse pre-emulsion. After 1 h, the mixture was then ultrasonicated (120 W, XO-SM50, Nanjing Xianou Instruments Manufacture Co., Ltd., Nanjing, China) for 5 min in an ice bath to afford a miniemulsion. Subsequently, the miniemulsion was then treated with a crosslinker BIEE (0.01 g, 0.027 mmol) and then stirred at room temperature for 4 days to facilitate crosslinking. The product was then dialyzed against methanol (3 × 150 mL) using a cellulose membrane (MWCO 12–14 kDa) to remove chloroform, unreacted crosslinker, and water. Finally, the suspension was adjusted to contain 9 g of methanol and 1 g of water for the next step.

### 2.6. Synthesis of Pentacyanoferrate-Coordinated Polymeric Nanoparticles ([Fe(CN)_5_HN_3_]^3−^@Polymeric Nanocapsules)

To the previously prepared P4VP_82_-*b*-PDMAA_180_ nanocapsule suspension, sodium pentacyanoammineferroate(III) Na_3_ [Fe(CN)_5_HN_3_] salt (0.05 g, 0.184 mmol) was added at room temperature. Upon addition, the suspension immediately changed color from milky white to orange. The vial was then sealed and the suspension was stirred for 3 days at room temperature in the dark. During this period, the color gradually turned from orange to yellowish-brown. After reaction, the mixture was then dialyzed against methanol (3 × 150 mL) to remove unreacted Na_3_ [Fe(CN)_5_HN_3_] salt, which is insoluble in methanol. Finally, the dialyzed suspension was centrifuged at 1000 rpm for 2 min, and the brownish-yellow supernatant was collected as the product.

### 2.7. Synthesis of Prussian Blue-Loaded Polymer Nanocapsules (PB@Polymeric Nanocapsules)

To the resulting suspension (containing [Fe(CN)_5_HN_3_]^3−^@polymeric nanocapsules), 0.1 mL of a FeCl_3_·6H_2_O solution in methanol/water (5:1, *w*/*w*, 0.1 M, containing 1.62 mg or 0.010 mmol FeCl_3_) was added dropwise at room temperature under stirring. The color immediately changed from brownish-yellow to bluish-green, indicating the formation of Prussian Blue (PB). The resulting PB-containing polymeric nanocapsules were characterized by UV–Vis spectroscopy, FT-IR, TGA, and TEM.

## 3. Results and Discussion

### 3.1. Preparation of Polymeric Capsules

2-Cyano-2-propylbenzodithioate (CPDB) was employed as a reversible addition–fragmentation chain transfer agent to mediate the polymerization of 4-vinylpyridine (4VP), yielding a well-defined P4VP macro chain transfer agent (P4VP-CTA). This P4VP-CTA was then used to initiate the polymerization of *N*,*N*-dimethylacrylamide (DMAA), forming poly(4-vinyl pyridine)-block-poly (*N*,*N*-dimethylacrylamide)/P4VP-*b*-PDMAA diblock copolymers (Scheme S1). Molecular weights Mn, polydispersities (PDIs), and repeat units of both P4VP and P4VP-*b*-PDMAA were characterized and calculated by GPC and ^1^H NMR (Figures S1 and S2), with the data summarized in Table 1. The increase in Mn from a homopolymer to diblock copolymer confirmed successful chain extension. Both polymers exhibited narrow PDIs (<1.48), indicating controlled polymerization. For P4VP_82_-CTA, the degree of polymerization (DP = 82) was determined by comparing the integration of phenyl proton signals from the RAFT end-group (δ = 7.3–8.0 ppm) with that of pyridyl proton signals from the 4VP repeating units (δ = 8.1–8.6 ppm), as shown in Figure S2. For the block copolymer P4VP_82_-b-PDMAA_180_, the block composition was calculated by comparing the integration of pyridyl protons from the P4VP block (δ = 8.1–8.6 ppm) with that of dimethyl group protons from the PDMAA block (δ = 2.8–3.3 ppm). Based on these ^1^H NMR analyses, the number-average molecular weight (Mn) was calculated as 8831 g·mol^−1^ for P4VP_82_-CTA (DP × monomer molecular weight + end-group) and 26,651 g·mol^−1^ for P4VP_82_-*b*-PDMAA_180_. Notably, the ^1^H NMR-derived Mn values exceeded those from GPC due to the use of PMMA standards, which underestimate true molecular weights. The amphiphilic nature of P4VP_82_-*b*-PDMAA_180_ makes it a suitable stabilizer for oil-in-water (chloroform in water) miniemulsions. This is due to its hydrophobic P4VP_82_ block and hydrophilic PDMAA_180_ block, which interact compatibly with the chloroform and water phases, respectively [19,37].

**Scheme 1:**
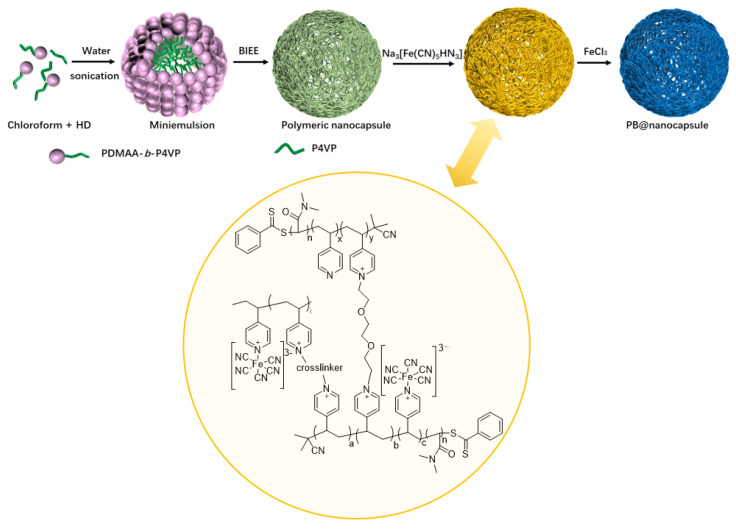
Schematic illustration for the preparation of PB-containing polymeric nanocapsules.

**Figure 1:**
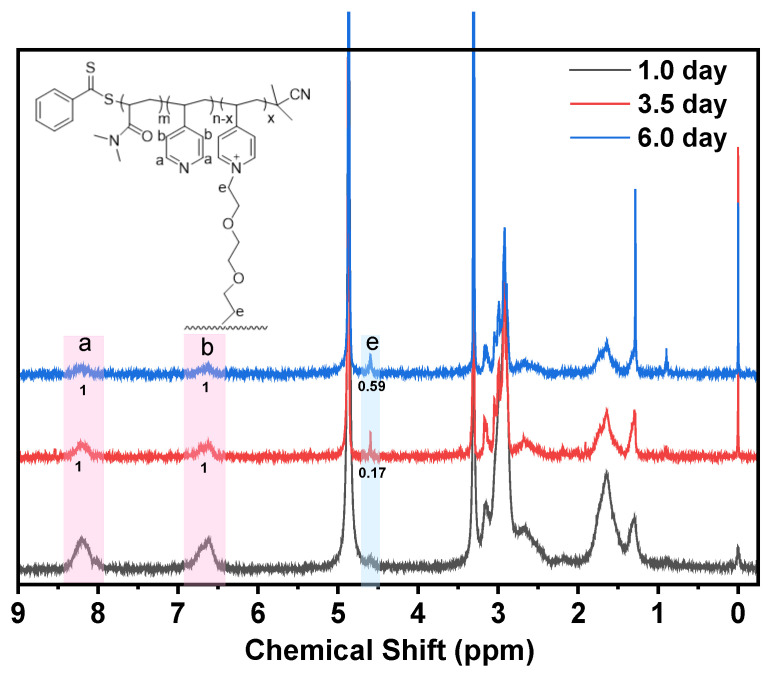
^1^H NMR spectra of the crosslinking reaction mixture taken after 1, 3.5, and 6 days in CD_3_OD; The letters a and b correspond to the hydrogen atoms on the pyridine rings, and e corresponds to the newly formed –N^+^–CH_2_– groups.

As illustrated in Scheme 1, the miniemulsion was formed from a chloroform, hexadecane(HD), P4VP_82_-*b*-PDMAA_180_ and water mixture in a weight ratio of 5:0.2:0.25:94.55. When varying amounts of P4VP homopolymer were added to different experimental groups, the composition of each component would be altered. In this system, the amphiphilic block copolymer P4VP_82_-*b*-PDMAA_180_ served as a stabilizer in a miniemulsion. The P4VP homopolymer serves as a hydrophobe for the miniemulsion, and also provides additional reactive sites for the subsequent binding of Prussian Blue to the polymeric capsules. To verify the hydrophobic nature of P4VP under our experimental conditions, the pH of the continuous phase was measured immediately after miniemulsion preparation. The pH was found to be in the range of 7–8. Under these neutral to slightly basic conditions, the P4VP blocks of the P4VP_82_-*b*-PDMAA_180_ and P4VP homopolymer remain non-protonated and therefore hydrophobic, as this pH range is well above the reported pKa values of P4VP (4.7) [38]. In addition, the pyridyl groups of the P4VP_82_-*b*-PDMAA_180_ and P4VP homopolymer could undergo a quaternization reaction with 1,2-bis(2-iodoethoxy)ethane (BIEE, crosslinker) to form the shell structure of the polymeric nanocapsule. The quaternization reaction of poly(4-vinylpyridine) enhances its water solubility [39]. This causes the P4VP homopolymer, originally located within the chloroform in the miniemulsion, to gradually migrate toward the oil–water interface of the miniemulsion as the quaternization proceeds, thereby further improving the stability of the miniemulsion. A 3–4-day crosslinking reaction is required to fix the nanocapsule structure at room temperature. To monitor the crosslinking process, small aliquots were taken on days 1, 3.5, and 6. Unreacted crosslinker and chloroform were removed by dialysis against methanol, and the dried samples were redissolved in CD_3_OD for ^1^H NMR analysis. As shown in Figure 1, with the increasing reaction time, a clear enhancement in the signal at δ 4.6 ppm is observed. This signal is attributed to the newly formed –N^+^–CH_2_– groups, confirming the occurrence of the crosslinking reaction. However, the anticipated downfield shift in the pyridine ring protons (to δ 8.8–9.2 ppm) due to quaternization of the adjacent nitrogen atom is not evident in the spectra. This absence can be explained by the reduced solubility of the crosslinked polymer in CD_3_OD, which has two consequences: (i) a highly crosslinked polymer (rich in pyridinium groups) precipitates and no longer contributes to the detectable NMR signal; (ii) the remaining swollen fraction suffers from restricted molecular mobility, leading to severe line broadening and loss of signals into the baseline. Therefore, we cannot directly monitor the pyridinium protons. Instead, we rely on the signal of the –N^+^–CH_2_– groups to represent the amount of pyridine that has undergone quaternization, and combine it with the signal of unreacted pyridine (δ 8.2 ppm) to calculate the quaternization ratio. After 3.5 days of reaction, the obtained quaternization ratio is approximately 14.5%. It should be noted, however, that the actual degree of crosslinking may be lower than this value, because some crosslinker molecules may have only one of their two iodine atoms participating in the quaternization reaction. Consistent with our observation, Armes and co-workers reported that the quaternization of poly(2-(dimethylamino)ethyl methacrylate) (PDMAEMA) with BIEE also proceeds slowly at room temperature, with only a low degree of crosslinking achieved after several days [40].

**Figure 2:**
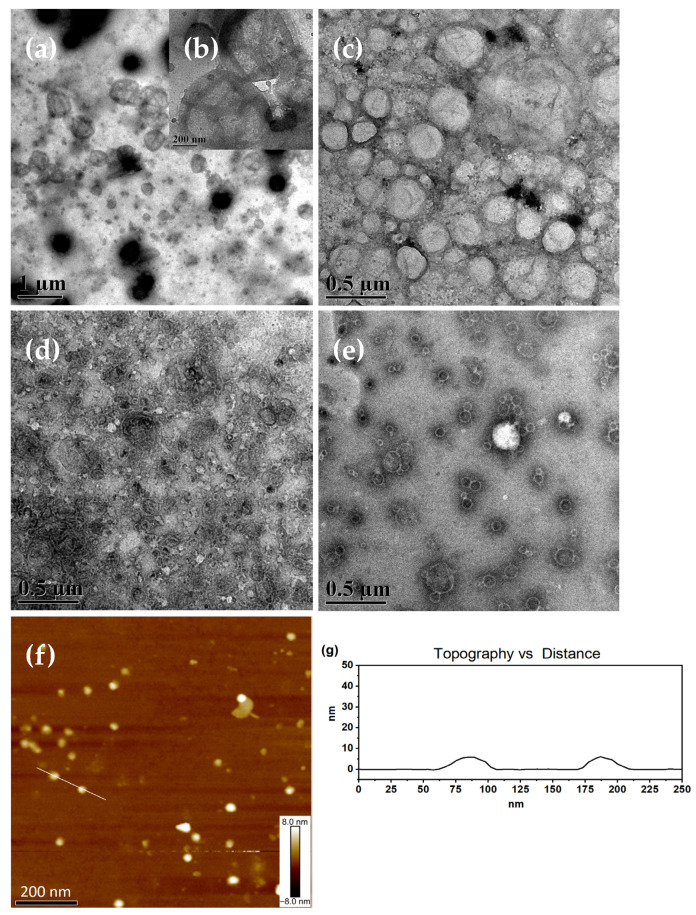
TEM images of nanocapsules were prepared by using P4VP_82_-*b*-PDMAA_180_ as stabilizers with varying amounts of P4VP homopolymer in the oil phase: (**a**): without P4VP homopolymer; (**b**) the same conditions as (**a**), except that the scale bar has been enlarged; (**c**): containing 0.003 g of P4VP homopolymer; (**d**): containing 0.005 g of P4VP homopolymer and (**e**) containing 0.01 g of P4VP homopolymer. All TEM samples were stained by phosphotungstic acid (2%); (**f**) AFM image obtained from a sample prepared under the same conditions as in (**d**); (**g**) topography vs. distance image of selected particles as highlighted in (**f**).

**Figure 3:**
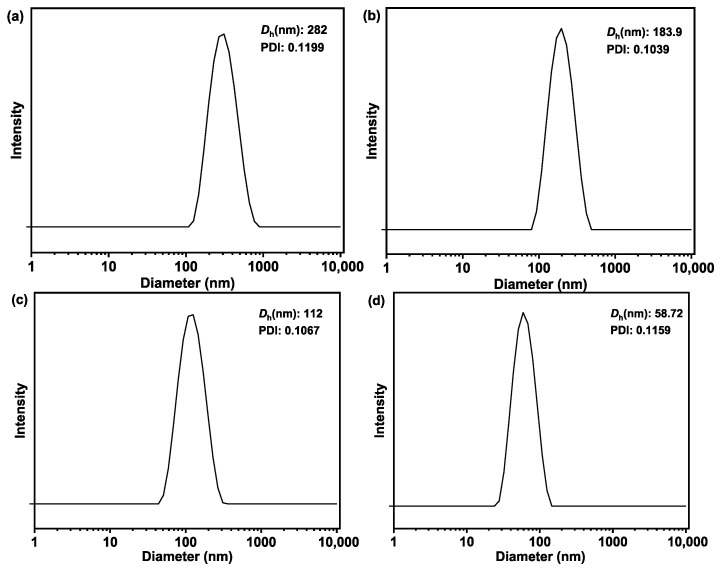
Diameter and polydispersity of nanocapsules prepared by using P4VP_82_-*b*-PDMAA_180_ as stabilizers with varying amounts of P4VP homopolymer in the oil phase: (**a**) without P4VP homopolymer; (**b**) containing 0.003 g of P4VP homopolymer; (**c**) containing 0.005 g of P4VP homopolymer and (**d**) containing 0.01 g of P4VP homopolymer. All samples were dispersed in DI water for measurement.

Since the pyridine groups in P4VP serve as active sites for the subsequent synthesis of Prussian Blue, this study first investigated the effect of adding varying amounts of P4VP homopolymer into the miniemulsion formulation on the morphology and size of the miniemulsion. The crosslinked nanocapsules without the addition of the P4VP homopolymer were first prepared and characterized by TEM (Figure 2a). The TEM sample was treated by phosphotungstic acid (2%) for negative staining. Here, flattened spherical particles with a diameter of approximately 200–700 nm were observed, indicating that the path we designed can yield polymer capsules. Similarly, the observed deflated ball-like structure (in Figure 2b) suggests that this type of polymer capsule is composed of a flexible thin film, which is consistent with the character of miniemulsion surfactant crosslinking. Although the particle size is not uniform, dynamic light scattering measurements revealed that most particle sizes were around 282 nm, as shown in Figure 3a. It can be observed that the capsule diameters measured by DLS and those observed by TEM show poor consistency. This may be attributed to the limited stability of the miniemulsion, which arises from the use of the P4VP_82_-*b*-PDMAA_180_ block copolymer as a stabilizer and HD as a hydrophobe—a combination that is not sufficient to suppress the Ostwald ripening process. As a result, the particle size becomes non-uniform, and it is precisely this non-uniformity that leads to the large deviation between the values obtained by TEM and DLS when determining the particle size. In the following experiments, P4VP homopolymers were incorporated into the oil phase of the miniemulsion as a hydrophobe, due to the good solubility of P4VP in chloroform. Consequently, the oil phase was supplemented with 0.003 g, 0.005 g, and 0.01 g of P4VP homopolymer individually, while maintaining consistency in other raw material compositions. As shown in Figure 2c–e, as the amount of P4VP homopolymer increases, the particle size decreases (consistent with DLS results in Figure 3a–d), and the vesicles also become more uniform in size. Especially in Figure 2e, the particle size is notably reduced to approximately 50 nm on average, with a concomitant significant decrease in the number of large capsules. This observation is roughly consistent with the dynamic light scattering (DLS) result of approximately 58 nm, as shown in Figure 3d, which is slightly larger than the TEM value because DLS measures the hydrodynamic diameter including the surface hydration layer in solution, whereas TEM images the dried, collapsed vesicle core under vacuum. The AFM image in Figure 2f further confirms the formation of spherical particles. Moreover, as shown in the topography–distance profile of selected particles (highlighted in Figure 2f) in Figure 2g, the selected particles exhibit a height of about 6 nm and a width ranging from 40 to 50 nm, yielding a low height-to-width ratio of approximately 0.12–0.15. This indicates that the nanocapsules collapsed upon drying, which is characteristic of soft spherical structures and consistent with the formation of polymeric nanocapsules [41]. A controlled set of experiments further demonstrated that adding P4VP homopolymer into the miniemulsion could enhance its stability. The miniemulsions without a crosslinking reaction were left standing after preparation to observe their phase separation behavior. The sample without P4VP homopolymer showed visible phase separation within 3–4 days. As the amount of P4VP homopolymer increased, the miniemulsion stability improved significantly. Phase separation occurred after about 6 days for the miniemulsion with 0.003 g of P4VP homopolymer, after about 10 days for that with 0.005 g, and only after approximately 15 days for the miniemulsion containing 0.01 g of P4VP homopolymer.

To further understand the stabilization mechanism, zeta potential measurements were performed on the miniemulsions (both before and after crosslinking) by dispersing 1 g of the sample into 10 g of water. As shown in Table S1, the zeta potential values consistently ranged from −1.0 to −1.5 mV, regardless of the amount of P4VP homopolymer added or whether crosslinking had been carried out. This near-neutral surface charge confirms that the stability of these miniemulsions does not rely on electrostatic repulsion, but rather on steric stabilization provided by the neutral PDMAA blocks [42,43]. The crosslinking reaction involves quaternization of the pyridine groups in P4VP, which could introduce positive charges. However, the zeta potential results suggest that such charges are either confined to the capsule interior or screened by the outer PDMAA layer. Therefore, the enhanced stability observed after crosslinking is not due to increased electrostatic repulsion, but likely arises from the structural reinforcement of the capsule shell [44]. Furthermore, the role of the P4VP homopolymer in this system is primarily as a hydrophobe. This is consistent with the observation that adding more P4VP homopolymer progressively improves the resistance to phase separation, as a higher amount of hydrophobe can suppress Ostwald ripening to a large extent and reduce the droplet size [45,46].

### 3.2. Synthesis and Characterization of Prussian Blue-Loaded Polymer Nanocapsules (PB@Polymeric Nanocapsules)

As schematically illustrated in Scheme 1, Prussian Blue-loaded polymer nanocapsules/PB@polymeric nanocapsules were synthesized via a miniemulsion crosslinking reaction. The obtained polymeric nanocapsules were then purified by dialysis against methanol to eliminate unreacted crosslinker, water, and chloroform. During dialysis, the miniemulsion gradually changed from a milky-white suspension into a more transparent, turbid liquid, and no precipitation was observed. These observations indicate that methanol effectively disrupted the oil–water equilibrium of the miniemulsion, and the large excess of methanol facilitated the removal of chloroform and unreacted crosslinker. The absence of precipitation further suggests that the degree of crosslinking was limited, which is consistent with the fact that the solubility of P4VP in methanol decreases as the degree of quaternization increases. After dialysis, the methanol content was adjusted to 9 g, followed by the addition of 1 g of water. Then, 0.05 g of Na_3_ [Fe(CN)_5_HN_3_] salt was added to generate pentacyanoferrate-coordinated polymeric nanoparticles ([Fe(CN)_5_HN_3_]^3−^@polymeric nanocapsules). Our previous studies have confirmed that the P4VP pyridyl moiety could react with Na_3_ [Fe(CN)_5_HN_3_] through ligand exchange [47]. Because the solubility of Na_3_ [Fe(CN)_5_HN_3_] in methanol is very low, a small amount of water was added in the previous step to ensure that sufficient salt remained available to react with the pyridine groups on the polymeric nanocapsules.

To further verify the feasibility of the experimental design, we performed a kinetic study on the ligand exchange reaction between the polymeric nanocapsule and Na_3_ [Fe(CN)_5_HN_3_]. As shown in Figure S3, with the progress of the reaction, the absorption intensity due to the coordination between pentacyanoferrate complexes and pyridyl groups increases gradually, indicating that the coordination reaction occurs [19].

**Figure 4:**
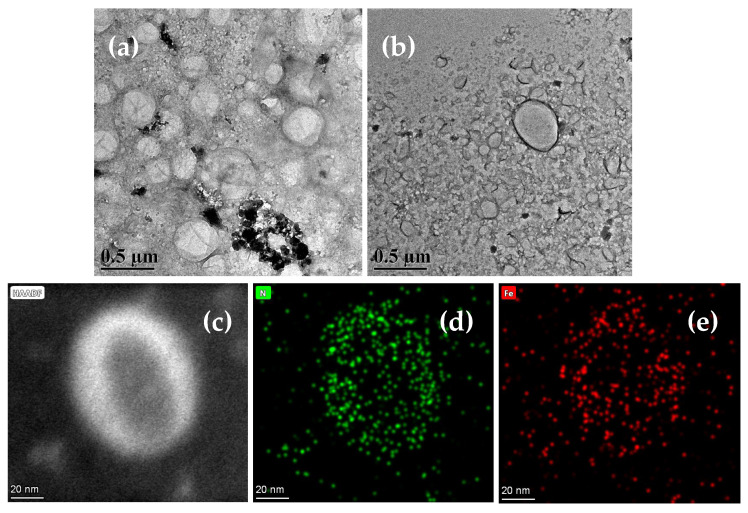
TEM images of samples prepared from PB@polymeric nanocapsules without (**a**) and with 0.01 g of P4VP homopolymer (**b**) at lower magnification; (**c**) HAADF-STEM image of the same sample at higher magnification; and (**d**) EDX mapping of nitrogen and (**e**) mapping of iron element.

Throughout both the coordination reaction and the subsequent removal of unreacted salt, the polymeric capsules remain stably suspended in the solution without precipitation. After the coordination reaction, the suspension was dialyzed against methanol to remove water and precipitate the unreacted salt. The resulting mixture was treated with an FeCl_3_∙6H_2_O solution (0.1 M) in methanol/water, which induced an immediate color change from orange to blue, suggesting the formation of Prussian Blue Fe_4_ [Fe(CN)_6_]_3_ via coordination between the [Fe(CN)_5_L]^3−^ ligands and Fe^3+^ ions. Both the polymeric nanocapsules prepared with and without P4VP homopolymer (0.01 g) were utilized in an attempt to synthesize PB@polymeric nanocapsules. The two samples were first examined by TEM to observe the morphology of the capsules after Prussian Blue loading. During TEM sample preparation, no counterstaining treatment (with phosphotungstic acid) was applied. The sample without P4VP homopolymer is shown in Figure 4a. The deflated capsule structure can still be observed. This is because the Prussian Blue has attached to the polymer capsule, enhancing the electron density of the polymer nanocapsules. However, the contour and structure of the polymer capsules are not clearly resolved, appearing less distinct compared to the nanocapsules shown in Figure 2a. (Note: The polymer capsules in Figure 2a were subjected to phosphotungstic acid staining.) This observation can be attributed to the dual role of the pyridyl groups in the P4VP_82_-*b*-PDMAA_180_ copolymer used for nanocapsule preparation, which are involved in both the crosslinking reaction and the formation of Prussian Blue. The limited amount of copolymer restricts the in situ generation of Prussian Blue, resulting in low electron density contrast between the polymer capsules and the background in TEM images (Figure 4a). Meanwhile, the distribution of Prussian Blue across capsules of different sizes appears non-uniform. In contrast, the polymer capsules that were prepared containing 0.01 g of P4VP homopolymer exhibited a better display of their structure after being coated with Prussian Blue, as depicted in Figure 4b. This is because the pyridyl group of the P4VP homopolymer on nanocapsules enhances the coordination reaction between the overall polymer capsule and the Na_3_ [Fe(CN)_5_HN_3_] salt, allowing for more in situ generation of Prussian Blue on the polymer nanocapsule. The particles exhibit a collapsed, ring-like morphology, characteristic of a hollow shell structure [30]. The identical sample was further analyzed by HAADF-STEM, as shown in Figure 4c–e. As shown, the elemental mappings of N and Fe exhibit a good overlap, demonstrating that iron is uniformly distributed on the nanocapsules, consistent with the polymer template. This further indicates that Prussian Blue can be uniformly formed in situ on the polymer nanocapsules. For further characterization and study, Prussian Blue-loaded polymer nanocapsules (PB@polymeric nanocapsules) prepared with 0.01 g of P4VP homopolymer were used unless otherwise indicated.

**Figure 5:**
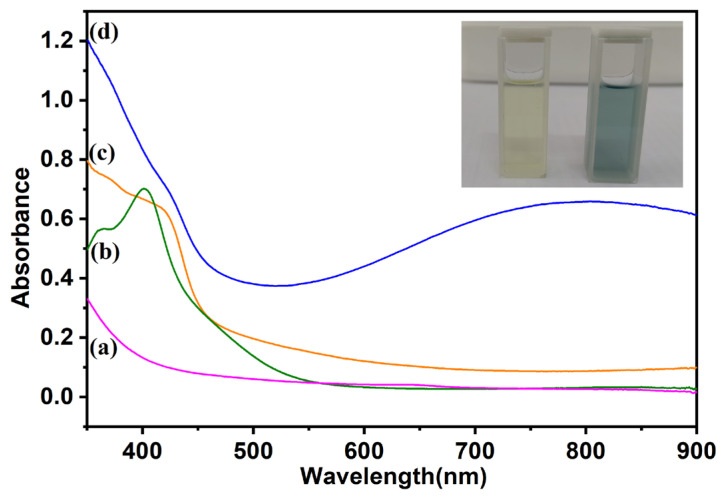
UV–Vis spectra of (**a**) crosslinked nanocapsules, (**b**) Na_3_ [Fe(CN)_5_HN_3_], (**c**) [Fe(CN)_5_HN_3_]^3−^@polymeric nanocapsules and (**d**) PB@polymeric nanocapsules. Inset shows digital photos of [Fe(CN)_5_HN_3_]^3−^@nanocapsules (**left**) and after (**right**) addition of Fe^3+^.

UV–Vis spectroscopy was employed to characterize the polymeric nanocapsules, Na_3_ [Fe(CN)_5_HN_3_], [Fe(CN)_5_HN_3_]^3−^@polymeric nanoparticles and PB@polymeric nanocapsules. Prior to analysis, the Fe(CN)_5_HN_3_]^3−^@polymeric nanoparticles and PB@polymeric nanocapsules were prepared by mixing 1.0 g of each suspension into 10 g of a methanol/water solution (9:1, *w*/*w*). As shown in Figure 5, the polymeric nanocapsules without metal complexes exhibited no absorption in the visible range. The free Na_3_ [Fe(CN)_5_HN_3_] complex and the [Fe(CN)_5_HN_3_]^3−^@polymeric nanocapsules show their main charge transfer absorption in the near-UV/blue region (ca. 400–450 nm), as reported by Jannuzzi et al. [48]. Therefore, only the PB@polymeric nanocapsules display a broad absorption peak in the 600–900 nm range, attributed to the charge transfer between Fe(II) and Fe(III) through cyanide bridges in the resulting Prussian Blue [47]. This confirms that the characteristic visible absorption of Prussian Blue appears only after the formation of the PB structure.

**Figure 6:**
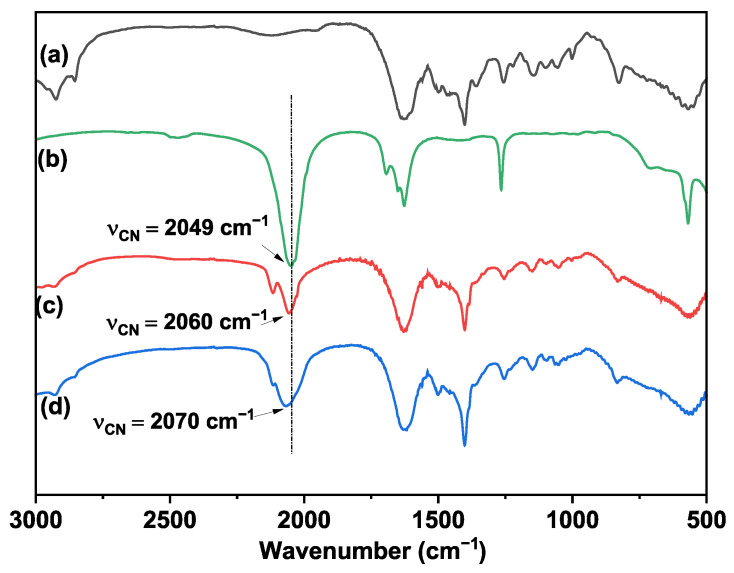
FT-IR spectra of (**a**) polymeric nanocapsules, (**b**) Na_3_ [Fe(CN)_5_HN_3_], (**c**) [Fe(CN)_5_HN_3_]^3−^@nanocapsules, (**d**) PB@nanocapsules.

Further evidence for the formation of [Fe(CN)_5_HN_3_]^3−^@polymeric nanoparticles and PB@polymeric nanocapsules was obtained from FT-IR spectroscopy. As shown in Figure 6a,b, the spectra correspond to the crosslinked polymer capsules and Na_3_ [Fe(CN)_5_NH_3_], respectively. The CN stretching vibration peak of Na_3_ [Fe(CN)_5_HN_3_] appears at 2049 cm^−1^. After coordination between the pyridine groups in the crosslinked capsules and Na_3_ [Fe(CN)_5_HN_3_], the CN stretching vibration of the resulting complex shifts to 2060 cm^−1^, indicating a blue shift, as shown in Figure 6c. This blue shift arises from competition between the pyridyl groups and the CN ligands for the d electrons of the Fe centers. This competition weakens the п-back donation from Fe to CN, thereby reducing the electron density in the CN antibonding orbitals. Consequently, the CN bond order increases, leading to a stronger CN bond. Thus, the blue shift in the CN stretching mode unambiguously confirms the attachment of the ferrate complexes to the pyridyl groups [19]. Upon introduction of Fe^3+^, a noticeable blue shift in the CN stretching band from 2060 to 2070 cm^−1^ is detected in Figure 6d, indicating a cyanide bridge formation between Fe(II) and Fe(III), in agreement with previous studies on Prussian Blue systems [49].

**Figure 7:**
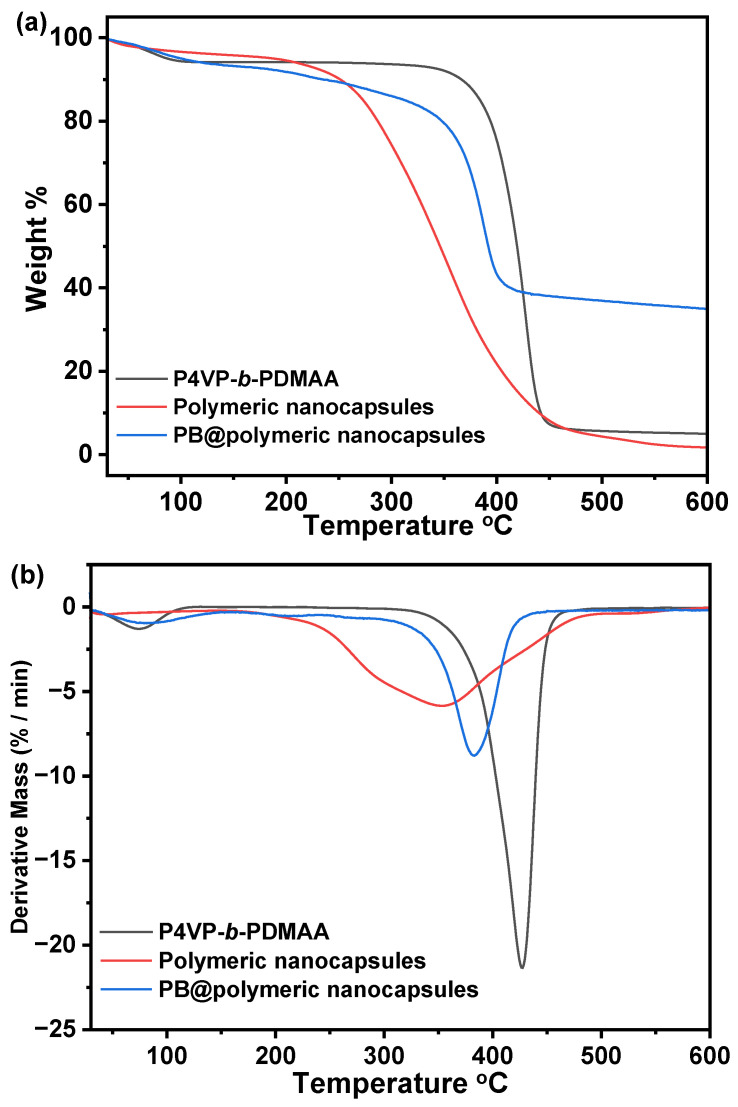
TGA (**a**) and DTG (**b**) traces of P4VP_82_-*b*-PDMAA_180_, polymeric nanocapsules and PB@polymeric nanocapsules.

The thermal degradation behavior of the block copolymer P4VP_82_-*b*-PDMAA_180_, polymeric nanocapsules, and PB@polymeric nanocapsules was assessed through thermogravimetric analysis (Figure 7). The P4VP_82_-*b*-PDMAA_180_ exhibits two stages of mass loss. This is evidenced by both the TGA and DTG curves. The first minor loss occurring below 150 °C is associated with the removal of adsorbed water or other residual solvents under inert conditions. This is reflected in the DTG trace as a small shoulder at low temperature. The main degradation event, observed between 400 °C and 450 °C, corresponds to the thermal decomposition of the block polymer backbone, and the DTG curve shows a sharp peak at approximately 430 °C, indicating a well-defined maximum degradation rate. In contrast, the polymeric nanocapsules degrade at lower temperatures, with the major mass loss occurring between 250 °C and 500 °C. The DTG curve of the nanocapsules reveals a distinct degradation peak around 350 °C, confirming their reduced thermal stability compared to the pristine block copolymer. This lower stability is attributed to the facile loss of ammonium groups from quaternized P4VP via Hofmann elimination [50]. Notably, the deposition of PB on the polymeric capsules (PB@polymeric nanocapsules) significantly enhances thermal stability. The TGA trace of PB@polymeric nanocapsules shifts to higher temperatures, and the corresponding DTG peak moves to approximately 370 °C. This observation strongly supports that the formation of a PB layer on the polymer effectively protects the underlying nanocapsules from thermal decomposition during heating.

### 3.3. Vary Amount of PB on the Polymeric Nanocapsules

During our investigation, we attempted to regulate the loading amount of Prussian Blue (PB) on the polymeric capsules by controlling the amount of Fe^3+^ introduced. To ensure a consistent concentration of the [Fe(CN)_5_HN_3_]^3−^@polymeric nanocapsule suspension for the reactions with ferric ions in this series of experiments, the reagent amounts specified in Section 2.5 and Section 2.6 were scaled up by a factor of five during preparation. The resulting suspension was then evenly divided into five portions, each weighing 10 g and containing approximately 17.203 mmol of pyridine groups. Among these pyridine groups, some participated in the crosslinking reaction, while the others were involved in coordination with [Fe(CN)_5_HN_3_]^3−^. To each 10 g portion of the suspension, Fe^3+^ was added in varying quantities: 5.0 × 10^−3^, 1.0 × 10^−2^, 2.0 × 10^−2^, 3.0 × 10^−2^, and 4.5 × 10^−2^ mmol, respectively. The resulting PB@polymeric nanocapsules were characterized using TEM, UV–Vis spectroscopy and TGA.

**Figure 8:**
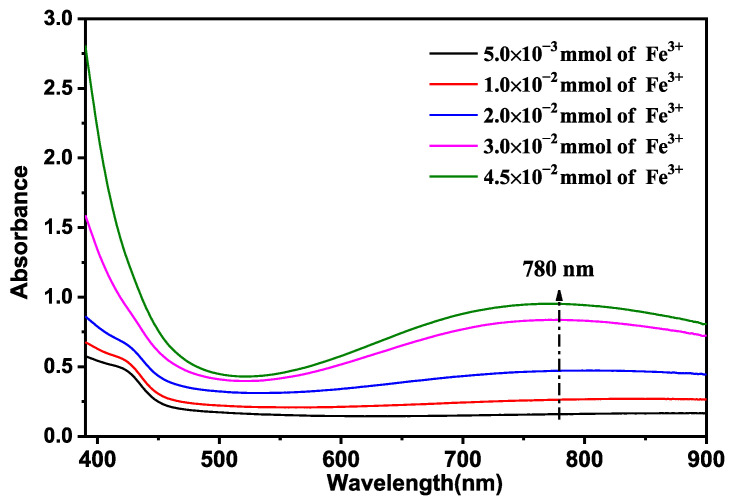
UV–Vis spectra of PB@polymeric nanocapsules in this set consist of 5.0 × 10^−3^ mmol, 1.0 × 10^−2^ mmol, 2.0 × 10^−2^ mmol, 3.0 × 10^−2^ mmol and 4.5 × 10^−2^ mmol of Fe^3+^.

All PB@polymeric nanoparticle samples require dilution prior to UV measurement. Consistent with the formation of Prussian Blue observed in Figure 4, the UV–Vis spectra of the PB@polymeric nanocapsules in Figure 8 also exhibit a distinct absorption peak at about 780 nm, corresponding to Prussian Blue. Prior to measurement, all PB@polymeric nanocapsule samples were diluted by mixing 1.0 g of suspension into 10 g of a methanol/water solution (9:1, *w*/*w*). As shown in Figure 8, with increasing amounts of added Fe^3+^, the absorption peak intensity increases significantly, indicating that the amount of PB produced also increases. However, when the Fe^3+^ amount exceeds the range of 3.5 × 10^−2^–4.5 × 10^−2^ mmol, the solution loses homogeneity and forms abundant blue precipitate. Hence, the maximum Fe^3+^ addition was set at 4.5 × 10^−2^ mmol, and this precipitation threshold defines an effective saturation region for PB loading in this system.

**Figure 9:**
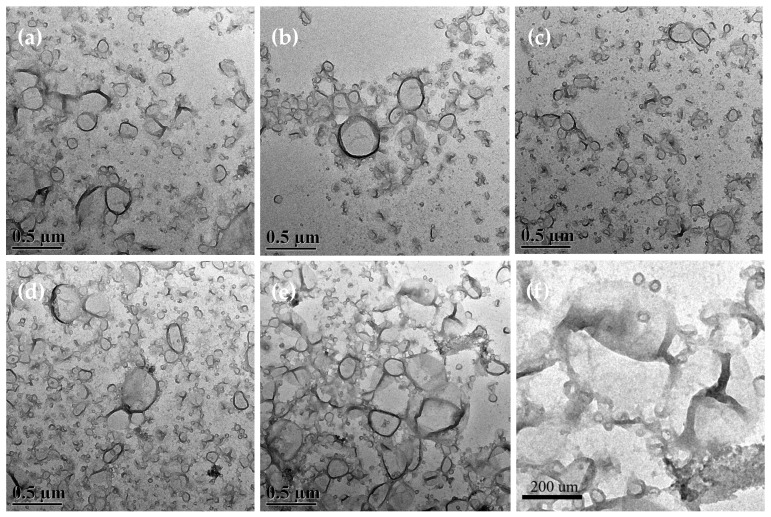
TEM images of PB@polymeric nanocapsules in this set consist of (**a**) 5.0 × 10^−3^ mmol, (**b**) 1.0 × 10^−2^ mmol, (**c**) 2.0 × 10^−2^ mmol, (**d**) 3.0 × 10^−2^ mmol and (**e**) 4.5 × 10^−2^ mmol of Fe^3+^; (**f**) the same conditions as (**e**), except that the scale bar has been enlarged.

The TEM analysis revealed that the capsule structure in Figure 9e exhibited enhanced clarity compared to that in Figure 9a as Fe^3+^ gradually increased. Figure 9e presents a magnified view of the sample depicted in Figure 9f, unveiling a distinct ring structure formed by polymer capsules with indentations. Moreover, as the number of stacked layers increases, there is a noticeable intensification in the darkness along the boundary of the capsule. This phenomenon can be attributed to the fact that an increased amount of PB within the capsule leads to a higher density and reduced light transmission. As a result, the contrast between the capsule and its background is enhanced.

**Figure 10:**
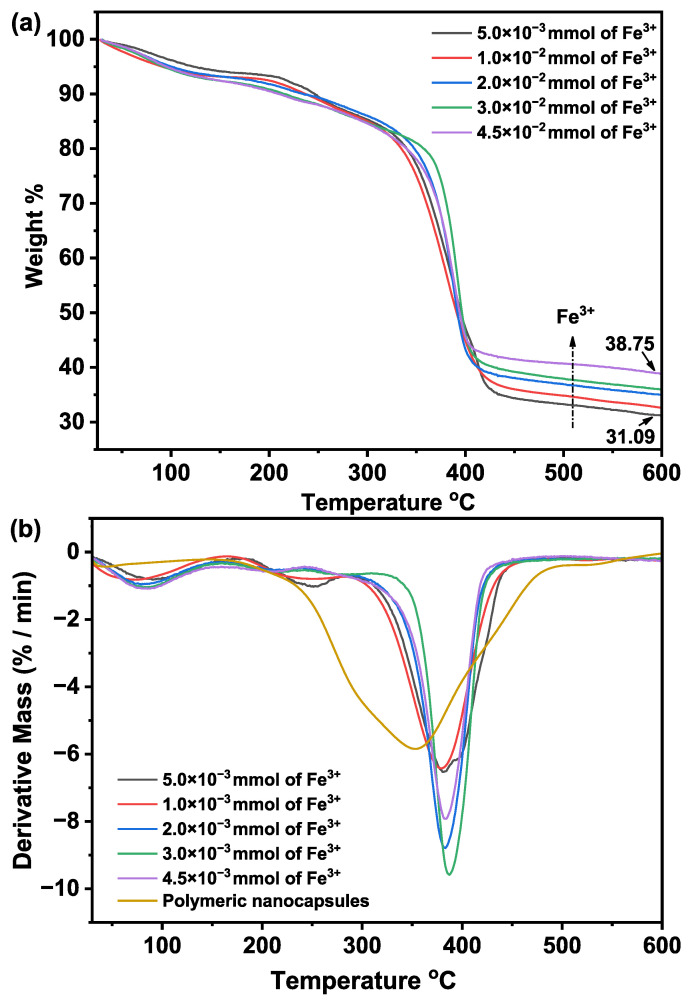
TGA (**a**) and DTG (**b**) traces of PB@polymeric nanocapsules with various amounts of Fe^3+^.

The TGA and DTG data in Figure 10 collectively illustrate the effect of Fe^3+^ addition (resulting in different amounts of Prussian Blue coating) on the thermal degradation behavior of PB@polymeric nanocapsules, in comparison with the pristine polymeric nanocapsules. For all the samples containing PB (those prepared with Fe^3+^ addition), a slight weight loss below 250 °C is observed in the TGA curves (Figure 10a), which is attributed to the removal of coordinated or crystalline water from the Prussian Blue structure [51]. This interpretation is evidenced by the DTG curves (Figure 10b). Only the PB-bearing samples display a distinct derivative peak between 100 and 200 °C, whereas the pure polymeric nanocapsules lack this peak, confirming the role of PB dehydration. Furthermore, combining the TGA and DTG profiles (Figure 10a,b), the main degradation of the PB-containing polymeric nanocapsules occurs between 300 and 450 °C. Notably, as the iron content (i.e., the amount of Prussian Blue) increases, the temperature range of this major degradation becomes distinctly narrower, indicating a more concentrated thermal decomposition process. This narrowing effect is particularly pronounced when compared with the polymeric nanocapsules, which lack Prussian Blue and exhibit a broader degradation temperature window. The pristine polymeric nanocapsules display a relatively broad degradation peak centered at approximately 350 °C, whereas the PB-coated samples show a progressive shift in the degradation peak to higher temperatures with increasing PB loading, accompanied by a reduction in the peak width. The TGA data (Figure 10a) show a significant increase in residue mass, which is consistent with an increased iron content arising from the formation of a PB coating, though the residue mass cannot be attributed exclusively to PB decomposition due to possible complex interactions between PB and the polymer matrix. These observations suggest that the Prussian Blue layer not only enhances thermal stability but also promotes a more uniform degradation pathway.

## 4. Conclusions

This study demonstrates a straightforward yet effective two-stage strategy for fabricating polymeric nanocapsules loaded with Prussian Blue (PB). In the first stage, well-defined nanocapsules with readily tunable sizes were engineered via BIEE quaternization crosslinking of a P4VP_82_-*b*-PDMAA_180_-stabilized miniemulsion, utilizing a P4VP homopolymer as a versatile size-regulating agent. The second stage involved the precise incorporation and in situ formation of PB on the nanocapsule walls through sequential coordination with Na_3_ [Fe(CN)_5_HN_3_] and FeCl_3_, where the PB loading was conveniently controlled by the FeCl_3_ concentration. Overall, this work provides a robust and controllable platform for constructing PB-based functional nanocarriers, holding potential for applications in electrochemical fields such as sensing or energy storage.

## Figures and Tables

**Table 1:** Characteristics of the P4VP and P4VP-*b*-PDMAA block copolymers.

Polymer ^(a)^	M_theo._ (g mol^−1^)	M_n_ (g mol^−1^) ^(b)^	PDI ^(b)^
P4VP_82_-CTA	8831	7200	1.24
P4VP_82_-*b*-PDMAA_180_	26,651	18,900	1.48

^(a)^ The subscript number denotes the degree of polymerization (DP); ^(b)^ molecular weight and polydispersities (PDIs) of polymers were determined by GPC in Figure S1. The GPC system was calibrated with PMMA standards.

## Data Availability

The raw data supporting the conclusions of this article will be made available by the authors on request.

## Supplementary Materials

The following supporting information can be downloaded at: https://www.mdpi.com/article/10.3390/nano16090541/s1, Scheme S1: synthetic scheme for the preparation of P4VP-CTA and P4VP_82_-*b*-PDMAA_180_; Figure S1: GPC curves of the P4VP-CTA, P4VP_82_-*b*-PDMAA_180_ block copolymers; Figure S2: ^1^H NMR spectrum of P4VP-CTA and P4VP_82_-*b*-PDMAA_80_ block copolymers; Figure S3: UV–Vis spectra of the samples taken from the suspension of polymeric nanocapsules and Na_3_ [Fe(CN)_5_HN_3_] at different reaction times. (The sample preparation conditions were the same as those described in Section 2.6. Then, 1 g of the freshly prepared sample was taken and diluted with 9 g of an alcohol/water mixture for UV measurement); Table S1: summary of zeta potential measurements for different miniemulsion samples.

## Abbreviations

The following abbreviations are used in this manuscript:

CPDB	2-Cyano-2-propylbenzodithioate
P4VP	Poly(4-vinyl pyridine)
P4VP-CTA	P4VP macro chain transfer agent
P4VP-*b*-PDMAA	Poly(4-vinylpyridine)-block-poly(*N*,*N*-dimethylacrylamide)
HD	Hexadecane
BIEE	1,2-Bis(2-iodoethoxy)ethane
PB	Prussian Blue
[Fe(CN)_5_HN_3_]^3−^@polymeric nanocapsules	Pentacyanoferrate-coordinated polymeric nanocapsules
PB@polymeric nanocapsules	Prussian Blue-loaded polymer nanocapsules
