# Supplementary material for: Synthesis of Prussian Blue-Containing Polymeric Nanocapsules via Interfacial Confined Coordination in Crosslinked Miniemulsion
Source: Nanomaterials (Basel). 2026 Apr 29;16(9):541. doi: 10.3390/nano16090541 (PMC13164786; doi:10.3390/nano16090541)
Supplement: Supplementary file 1 [file nanomaterials-16-00541-s001.zip › nanomaterials-4243327-supplementary.pdf]

Synthesis of Prussian Blue-Containing Polymeric Nanocapsules via Interfacial Confined Coordination in Crosslinked Miniemulsion

Lin Wu 1,, Yubin Zhou 1, Tao Pang 2, Laxia Wu 2 and Yebin Guan 1,**

1 Anhui Ultra High Molecular Weight Polyethylene Fiber Engineering Research Center, School of Chemistry and Chemical Engineering, Anqing Normal University, Anqing 261433, China

2 Anhui Provincial Key Laboratory of Advanced Catalysis and Energy Materials, School of Chemistry and Chemical Engineering, Anqing Normal University, Anqing 261433, China

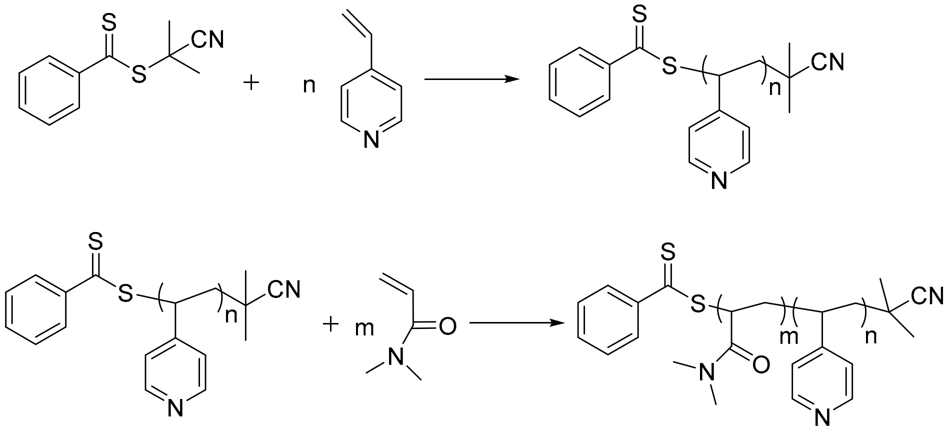

Scheme S1. Synthetic scheme for the preparation of P4VP-CTA and P4VP82-*b*-PDMAA$_{180}$.

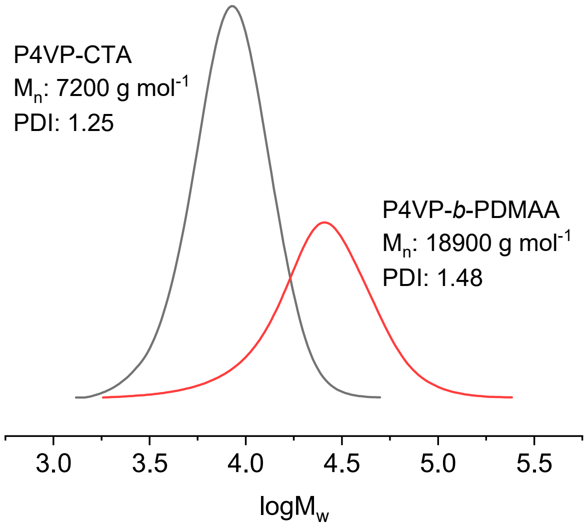

Figure S1: GPC curves of the P4VP-CTA, P4VP$_{82}$-*b*-PDMAA$_{180}$ block copolymers.

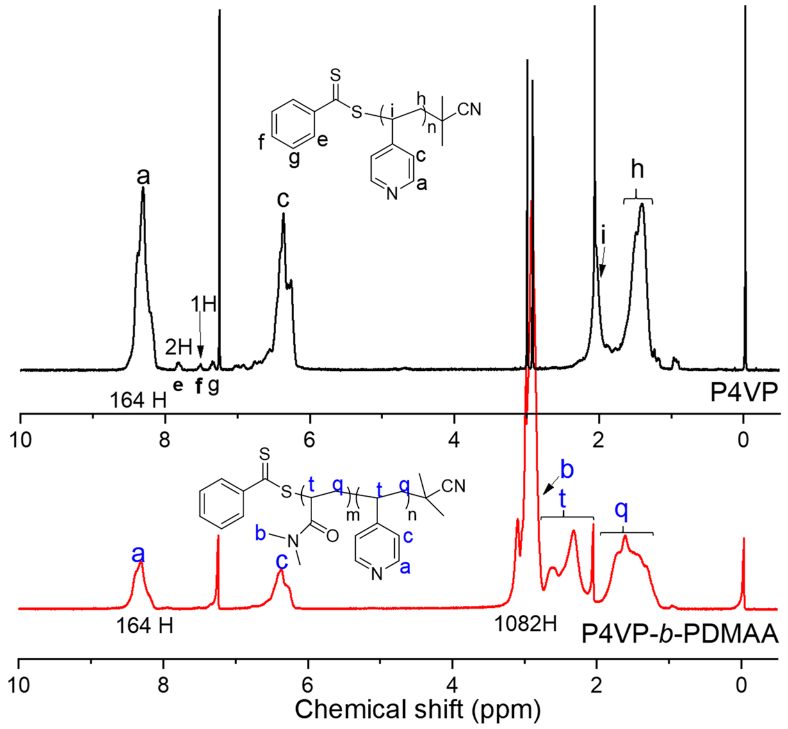

Figure S2:^{1}H NMR spectrum of P4VP-CTA and $P4VP_{82}$-*b*-$PDMAA_{180}$ block copolymers.

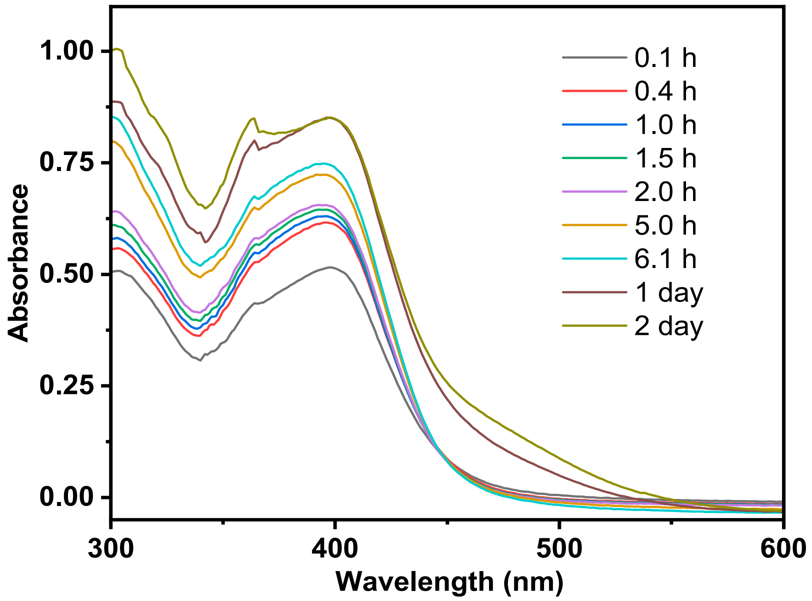

Figure S3. UV-Vis spectra of the samples taken from the suspensioin of polymeric nanocapsules and $Na_3[Fe(CN)_5HN_3]$ at different reaction times. (The sample preparation conditions were the same as those described in Section 2.6. Then, 1 g of the freshly prepared sample was taken and diluted with 9 g of an alcohol/water mixture for UV measurement.)

Table S1.Summary of zeta potential measurements for different miniemulsion samples.

Sample	The basic formulation	Amount of added P4VP (g)	Crosslinking status	Zeta (mV)			
				Run 1	Run 2	Run 3	Average
1	P4VP-*b*-PDMAA:0.025 g, BIEE: 0.01g HD:20 mg, Chloroform: 0.5 g, DI water:9.45 g	0	NO	-1.14	-1.17	-0.90	-1.07
2		0.003	NO	-1.70	-1.53	-1.06	-1.43
3		0.005	NO	-1.18	-0.90	-0.81	-1.01
4		0.01	NO	-1.71	-1.07	-0.81	-1.20
5		0.01	YES	-1.3	-1.55	-1.56	-1.47
